# Association between Ambient Temperature and Acute Myocardial Infarction Hospitalisations in Gothenburg, Sweden: 1985–2010

**DOI:** 10.1371/journal.pone.0062059

**Published:** 2013-04-30

**Authors:** Janine Wichmann, Annika Rosengren, Karin Sjöberg, Lars Barregard, Gerd Sallsten

**Affiliations:** 1 Department of Occupational and Environmental Medicine, Sahlgrenska University Hospital and University of Gothenburg, Gothenborg, Sweden; 2 Department of Molecular and Clinical Medicine, Sahlgrenska University Hospital and University of Gothenburg, Gothenborg, Sweden; 3 IVL Swedish Environmental Research Institute, Gothenborg, Sweden; University of Sao Paulo, Brazil

## Abstract

Cardiovascular disease (CVD) is the number one cause of death globally and evidence is steadily increasing on the role of non-traditional risk factors such as meteorology and air pollution. Nevertheless, many research gaps remain, such as the association between these non-traditional risk factors and subtypes of CVD, such as acute myocardial infarction (AMI). The objective of this study was to investigate the association between daily ambient temperature and AMI hospitalisations using a case-crossover design in Gothenburg, Sweden (1985–2010). A secondary analysis was also performed for out-of-hospital ischemic heart disease (IHD) deaths. Susceptible groups by age and sex were explored. The entire year as well as the warm (April−September) and cold periods (October–March) were considered. In total 28 215 AMI hospitalisations (of 22 475 people) and 21 082 out-of-hospital IHD deaths occurred during the 26-year study period. A linear exposure-response corresponding to a 3% and 7% decrease in AMI hospitalisations was observed for an inter-quartile range (IQR) increase in the 2-day cumulative average of temperature during the entire year (11°C) and the warm period (6°C), respectively, with and without adjustment for PM_10_, NO_2_, NO_x_ or O_3_. No heat waves occurred during the warm period. No evidence of an association in the cold period nor any association between temperature and IHD deaths in the entire year, warm or cold periods - with and without adjusting for PM_10_, NO_2_, NO_x_ or O_3_ was found. No susceptible groups, based on age or sex, were identified either. The inverse association between temperature and AMI hospitalisations (entire year and warm period) in Gothenburg is in accordance with the majority of the few other studies that investigated this subtype of CVD.

## Introduction

Cardiovascular disease (CVD) is the number one cause of death globally and also in European countries, such as Sweden [1]. Ischemic heart disease (IHD) is responsible for 61% of the 2 million CVD deaths in Europe annually, and stroke accounts for most of the rest. Acute myocardial infarction (AMI) is the most important manifestation of ischemic heart disease.

The biggest risk factors for CVD are age, sex, smoking, low physical activity, increased waist circumference, diabetes and hypertension [2]–[4]. Evidence is increasing on the effects of non-traditional risk factors, such as air pollution and weather (e.g. temperature) changes on CVD mortality and morbidity, specifically as short-term risk factors [4]–[6]. Nevertheless, fewer studies focused on subtypes of CVD, such as AMI hospitalisations.

The objective of this study was to investigate the association between daily ambient temperature and acute myocardial infarction (AMI) hospitalisations in a case-crossover design in Gothenburg, Sweden (1985–2010). A secondary aim was to study associations with out-of-hospital ischemic heart disease (IHD) deaths. Susceptible groups by age and sex were explored. We studied the entire year as well as warm (April–September) and cold periods (October–March). We addressed some of the limitations of the previous studies: investigated confounding by ambient air pollution (PM_10_, NO_2_, NO_x_ and O_3_), examined the lag and nonlinear effects of temperature and relative humidity, explored effect modification by age and sex by stratification and interaction models, and compared the results of the case-crossover models to those of the generalised additive Poisson time-series models.

## Methods

### Population and register data

Sweden has a publicly financed health care system with hospital care available to all citizens at low cost. Swedish hospitals register principal and contributory discharge diagnoses for all patients in the national hospital discharge register. For the purpose of the present study, data from the national hospital discharge register with coverage of all hospitalisations in Gothenburg since 1970 was used. The national cause of death register, based on diagnoses from death certificates, is complete from 1961.

#### Major coronary events and related case-fatality

The population for the present study includes all cases of AMI or fatal IHD events registered in Gothenburg from 1 January 1985 to 31 December 2010. The study period was determined by the availability of air pollution data. The International Classification of Diseases (ICD) version 9 (ICD-9) was used from 1987 to 1996 and version 10 (ICD 10) from 1997 and onwards.

Hospitalisation for an AMI was defined as a discharge (dead or alive) with a principal diagnosis of ICD 8–9 410 (until 1996); ICD 10 I21. Only emergency hospitalisations to any of the five hospitals within Gothenburg (about 1.7 to 5.8 km from the urban background station) were included in the study. In case AMI hospitalisations for the same person occurred at more than one hospital (i.e. transferrals) on a specific day, then only the first hospitalisation was retained in the study. AMI hospitalisations that occurred within 28 days after a previous AMI hospitalisation were excluded (3 068 admissions) as readmissions following discharge for AMI are quite high [7].

Out-of-hospital death was defined as a death from IHD (defined as ICD 8–9 410–414 (until 1996); ICD 10 I20–I25) in a person who was not hospitalised, as defined by not having a recorded admission on the day of death. Only deaths that occurred in Gothenburg were included in the study. The location of death was based on the home address of the deceased, but a minority might have died somewhere else.

### Air pollution data

Meteorological and air pollution data, except ground-level ozone (O_3_), were measured at the urban background monitoring station by the Gothenburg Environment and Health Department. The urban background monitoring station is located on the roof of a 25 m high building in the centre of Gothenburg with typical weekday traffic flows of about 65 000 vehicles/day in several directions (within 300 m) and minimal direct contribution from local pollution sources, in accordance with World Health Organisation (WHO) guidelines [8]. O_3_ was measured by IVL Swedish Environmental Research Institute on behalf of the Swedish Environmental Protection Agency and within the national air quality monitoring network at a rural background site located in Råö, 45 km south of the city centre.

Air pollution data included measurements of PM_10_ (tapered element oscillating microbalance instrument), nitrogen dioxides (NO_2_ and NO_x_) (chemiluminescence instrument) and O_3_ (UV absorption instrument). Please see Table S1 for the technical names of the various air pollution measurement instruments used during the study period. Temperature and relative humidity were measured with the HMP45a probe (Vaisala, Helsinki). Daily averages (midnight to midnight) data were applied in the statistical analyses. Missing values were not imputed. For 437 days the PM_10_ levels were lower than 5 µg.m^−3^ and were set as 5 µg.m^−3^.

### Ethics

Registry based health outcome data were applied in this study and all identifying variables were deleted. The study adheres to the standards of the Swedish Data Protection Agency. It was approved by the institutional review board of the University if Gothenburg which waived the need for written informed consent.

### Statistical analysis

The time-stratified case-crossover design was applied to investigate the association between temperature and AMI hospitalisations and IHD deaths. The case-crossover design was developed as a variant of the case-control design to study the effects of transient exposures on emergency events, comparing each person's exposure in a time period just prior to a case-defining event with person's exposure at other times [9]. Hereby, control on all measured and unmeasured personal characteristics that do not vary over a short time period is accomplished. If in addition, the control days are chosen close to the event day, personal characteristics that vary slowly over time (e.g. body mass index, smoking status) are also controlled by matching. A time-stratified approach was applied to select the control days, defining the day of hospitalisations or death as the case day and the same day of the other weeks in the same month and year as control days. With this approach even very strong confounding of exposure by seasonal patterns is controlled by design [10]–[12]. The data were analysed using conditional logistic regression analysis (PROC PHREG in SAS 9.2, SAS Institute, Cary, NC).

Lag0 (same day of exposure as day of hospitalisation or death) and lag1 (day prior to day of hospitalisation or death) were investigated, as well as the 2-day cumulative average (CA2) (mean of lag0–1). The values of CA2 were set as missing if any of the values of lag0 or lag1 were missing. Control days for lag1 and CA2 were defined as for lag0.

All models were adjusted for public holidays (as a binary variable). Previous studies reported a linear relationship between the air pollutants and AMI hospitalisations and CVD deaths [13]–[25]. The pollutants were therefore adjusted for as linear terms, one pollutant at a time.

Although intra-individual factors cannot be examined due to the nature of the case-crossover design where each person is his/her own control, inter-individual variation using an interaction term between the susceptibility variable and an exposure variable in the conditional logistic regression model yields the possibility to detect a p-value for interaction and when significant the subgroup specific estimates are valid. Susceptibility was therefore investigated in stratified analyses by sex and age (<75 and ≥75 years), followed by models with interaction terms.

Odds ratios (OR) and the 95% confidence intervals (CI) were calculated per inter-quartile range (IQR) increase in temperature and pollutant levels, which provide the magnitude-of-risk estimates that are comparable across the exposure variables. The results are presented as the percent excess risk in AMI hospitalisations or IHD deaths per IQR increase in a pollutant using the following calculation: (exp^(βxIQR)^−1)×100%, where β is the model estimate. For analysis of a given lagged exposure, a case was automatically dropped if exposure and meteorological data were not available for the case and at least one control day.

Sensitivity analyses were applied. The linearity of the relationship between AMI hospitalisations and the CA2 of temperature and the CA2 of relative humidity was confirmed in generalised additive Poisson time-series regression models (GAM) with the use of the *gam* procedure, *mgcv* package in R statistical software (R Development Core Team, 2010). We did not investigate this for the IHD deaths as no clear association with temperature was observed. The GAM design is the most suitable to explore the shape of an association. Its usefulness lies in the possibility of incorporating variables in a non-parametric way using smooth functions such as loess or spline, therefore avoiding the need to assume the shape of the association and later trying to reproduce it by means of an approximate functional expression. It also offers the opportunity to compare the results to that of the case-crossover analysis, i.e. investigate the robustness of the associations using different epidemiological study designs. Smoothing splines of calendar time with degrees of freedom per year (df/year) that varied from 0 to 5 were used to control for long-term trend and seasonality. The sum of the partial autocorrelation coefficients was minimised at 2.5 to 2.6 df/year, depending whether models were adjusted for PM_10_ or NO_2_ (CA2 lag) or none. The GAM models were also adjusted for day of the week and public holidays. Models were run with linear and non-linear terms of temperature and relative humidity, the latter as a smoothing spline function with 3 df. No difference in the results was observed by applying 4 or 5 df. We investigated whether the non-linear terms of temperature and relative humidity improved the models by conducting log-likelihood ratio tests. We decided not to use splines for temperature and relative humidity, as the splines were insignificant and did not add value to the models (Figure S1). As a linear dose-response was observed when data of the entire year were applied, GAM models were not run by the warm or cold period.

## Results

Table 1 indicates the characteristics of the 28 215 AMI hospitalisations (of 22 475 people) and 21 082 out-of-hospital IHD deaths during the study period. Of the 22 475 people who had AMI hospitalisations, 4 415 died at a later date out-of-hospital due to IHD. The mean age for AMI hospitalisations and out-of-hospital IHD deaths was 74 and 80 years, respectively. More hospitalisations and deaths occurred among men, those older than 75 years and during the cold period. Figures S2 and S3 illustrate the time-series of the daily number of AMI hospitalisations and IHD deaths during the study period. The number of AMI hospitalisations and the IHD deaths varied from 0 to 23 and 0 to 11 per day, respectively.

**Table 1:** Characteristics of the acute myocardial infarction hospital admissions and ischemic heart disease deaths in Gothenburg.

	Acute myocardial infarction hospital admissions during 1 January 1985 to 31 December 2010		Ischemic heart disease deaths during 1 January 1987 to 31 December 2010	
	No. cases	%	No. cases	%
**Total**	28215	100.0	21082	100.0
**Sex**				
Male	16627	58.9	11013	52.2
Female	11588	41.1	10069	47.8
**Age**				
23*–35 years	62	0.2	20	0.1
36–55 years	2459	8.7	659	3.1
56–65 years	3951	14.0	1488	7.1
66–75 years	7390	26.2	3913	18.6
76–84 years	9122	32.3	7012	33.3
85–102* years	5231	18.5	7990	37.9
**Period**				
Warm	13381	47.4	10149	48.1
Cold	14834	52.6	10933	51.9

* 25 years and 108 years for IHD deaths.

Table 2 provides an overview of the daily temperature, relative humidity and air pollution data. As expected, temperature levels were on average higher during the warm period (April–September). PM_10_ levels were on average similar during the warm and cold periods (October–March). Relative humidity, NO_2_ and NO_x_ levels were on average higher during the cold period, whilst this was the case for O_3_ levels during the warm period. The daily WHO and EU air quality limits (50 µg.m^−3^) for the measured PM_10_ levels were exceeded on 60 days at the urban background level during 1990–2010 (Figure S4) [8], [26]. On average, the temperature, relative humidity and air pollution levels on the case days were not significantly different from those on the control days (Table 2). No heat waves occurred during the 26-year study period. The Swedish Meteorological and Hydrological Institute defines a heat wave as those days when the daily maximum temperature is greater than 25°C for 5 days consecutively [27].

**Table 2:** Descriptive statistics for daily meteorological and air pollutant levels (lag0) in Gothenburg (1 January 1985–31 December 2010).

					Percentiles					
	No. days missing data	Mean	SD	Range	25^th^	50^th^	75^th^	IQR	AMI admissions Difference between case days and mean control days (95% CI)^a^	IHD deaths Difference between case days and mean control days (95% CI)^a^
**All year (9496 days)**										
Temperature (°C)	146	8.5	7.2	−22.0–26.2	3.3	8.4	14.4	11.1	0.0 (−0.1–0.0)	0.0 (0.0–0.1)
Relative humidity (%)	637	77.4	12.5	15.0–99.9	69.8	79.4	87.1	17.3	0.0 (−0.2–0.1)	0.1 (0.0–0.3)
PM_10_ (µg/m^3^)	2004	15.9	9.4	5–78.1	9.6	14.0	20.0	10.4	0.0 (−0.1–0.2)	−0.1 (−0.2–0.1)
NO_x_ (µg/m^3^)	739	58.2	71.2	4.4–1322.6	23.0	37.2	64.7	41.7	0.2 (−0.8–1.2)	−1.1 (−2.2– −0.1)
NO_2_ (µg/m^3^)	739	27.2	13.7	1.1–130.5	17.6	24.6	34.0	16.4	0.0 (−0.2–0.2)	−0.2 (−0.4–0.0)
O_3_ (µg/m^3^)	253	60.5	20.6	1.0–174.8	47.0	61.5	73.9	26.9	0.0 (−0.2–0.2)	0.1 (−0.1–0.3)
**Warm period (4758 days)**										
Temperature (°C)	47	13.8	4.6	−1.1–26.2	10.9	14.3	16.9	6.1	0.0 (−0.1–0.0)	0.0 (0.0–0.1)
Relative humidity (%)	304	72.2	12.3	32.0–99.0	64.7	73.5	81.2	16.5	0.0 (−0.3–0.2)	−0.1 (−0.3–0.2)
PM_10_ (µg/m^3^)	973	15.3	8.9	5–76.0	9.5	13.5	19.0	9.5	−0.1 (−0.2–0.1)	0.0 (−0.2–0.2)
NO_x_ (µg/m^3^)	435	42.5	36.1	4.4–520.6	20.6	31.6	51.6	31.0	−0.1 (−0.8–0.6)	−0.8 (−1.5–0.0)
NO_2_ (µg/m^3^)	435	24.0	11.2	1.1–102.3	15.8	21.9	30.1	14.3	0.0 (−0.2–0.2)	−0.2 (−0.4–0.0)
O_3_ (µg/m^3^)	90	71.3	17.0	14.5–174.8	60.3	70.8	81.6	21.2	−0.1 (−0.3–0.2)	0.2 (−0.2–0.5)
**Cold period (4738 days)**										
Temperature (°C)	99	3.1	5.0	−22.0–16.3	0.2	3.5	6.4	6.2	0.0 (−0.1–0.0)	0.0 (0.0–0.1)
Relative humidity (%)	333	82.7	10.3	15.0–99.9	77.4	84.8	90.1	12.7	0.0 (−0.2–0.1)	0.3 (0.1–0.5)
PM_10_ (µg/m^3^)	1031	16.4	9.8	5–78.1	9.7	14.6	20.8	11.1	0.1 (−0.1–0.3)	−0.2 (−0.4–0.0)
NO_x_ (µg/m^3^)	304	73.5	90.9	6.1–1322.6	26.7	44.7	81.5	54.8	0.5 (−1.2–2.2)	−1.5 (−3.3–0.3)
NO_2_ (µg/m^3^)	304	30.3	15.0	5.0–130.5	19.7	27.4	37.8	18.1	0.0 (−0.2–0.3)	−0.2 (−0.5–0.1)
O_3_ (µg/m^3^)	163	49.5	17.9	1.0–112.4	37.5	50.4	62.3	24.8	0.0 (−0.3–0.3)	0.0 (−0.3–0.3)

SD: Standard deviation.

IQR: Interquartile range.

a Differences between case days and control days are calculated by subtracting the average of the level on the associated control days from the case day. The average of these differences for the 28215 acute myocardial infarction admissions and 21082 ischemic heart disease deaths is then calculated.

Table 3 display the Spearman correlations between the daily temperature, relative humidity and air pollution levels during the warm and cold periods. Temperature had an inverse correlation with NO_2_ and NO_x_ during both periods - stronger during the cold period. Temperature had a stronger correlation with PM_10_ and O_3_ during the warm period. Relative humidity had an inverse correlation with temperature and the pollutants during both periods, except for temperature during the cold period. O_3_ had an inverse association with NO_2_ and NO_x_ - the strongest during the cold period. PM_10_ had a stronger correlation with the other pollutants during the warm period.

**Table 3:** Spearman correlation coefficients between exposure variables (daily lag0) in Gothenburg during the warm and cold periods (1 January 1985–31 December 2010).

Warm period	Temp	PM_10_	NO_x_	NO_2_	O_3_
**Rel. hum**	−0.161	−0.157	−0.144	−0.137	−0.315
	4454^a^	3781	4122	4122	4381
**Temp**		0.109	−0.098	−0.080	0.102
		3781	4319	4319	4623
**PM_10_**			0.120	0.234	0.364
			3555	3555	3777
**NO_x_**				0.920	−0.247
				4323	4249
**NO_2_**					−0.092
					4249

Cold period	Temp	PM_10_	NO_x_	NO_2_	O_3_
**Rel. hum**	0.183	−0.170	−0.126	−0.121	−0.502
	4405^a^	3664	4221	4221	4355
**Temp**		0.054^b^	−0.353	−0.348	0.032^b^
		3701	4428	4428	4480
**PM_10_**			0.042^b^	0.117	0.120
			3587	3587	3672
**NO_x_**				0.913	−0.279
				4434	4284
**NO_2_**					−0.162
					4284

a Number of days less than 4758 in the warm and 4738 in the cold period due to missing exposure data.

b p-value<0.05, otherwise p-value<0.0001.

**Figure 1:**
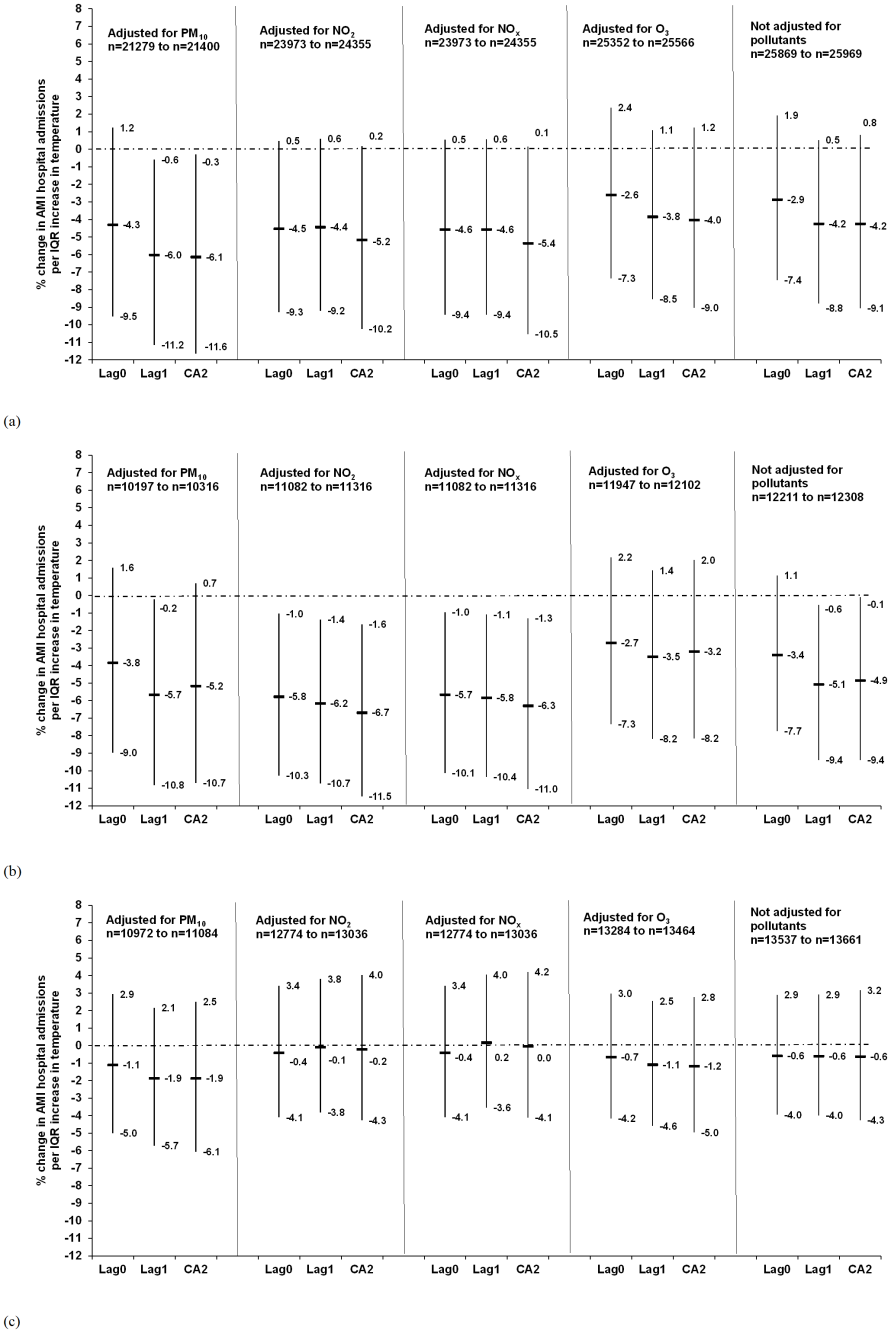
Association between temperature and acute myocardial infarction hospital admissions in Gothenburg, expressed as percentage increase in risk (%) and 95% confidence intervals per inter-quartile increase in daily lag0, lag1 and 2-day cumulative average during (a) the entire year, (b) warm period (April−September) and (c) cold period (October–March). Models adjusted for a single pollutant (same lag as temperature), relative humidity (same lag as temperature) and public holidays. Number of cases (n) used in the models is less than the original number due to missing exposure data.

Figure 1 and Figure S5 illustrate the % change in the AMI hospitalisations per IQR increase in the lags of temperature and the pollutants, respectively, after adjusting for public holidays and relative humidity in single pollutant models. The same lag of the pollutants, temperature and relative humidity was included in each model. None of the pollutants were significantly associated with AMI hospitalisations in the entire year, warm or cold period (Figure S5). However, the pollutants attenuated the association between temperature and AMI hospitalisations. In the entire year, an IQR increase in the CA2 of temperature was associated with an insignificant decrease of 4% (95% CI: −1%; 9%) in AMI hospitalisations without adjusting for any pollutant or adjusting for O_3_ (Figure 1). After adjusting for PM_10_, the association strengthened to −6% (95% CI: 0%; −12%) in the entire year. Adjusting for NO_2_ and NO_x_ resulted in a protective association of 5%, albeit not statistically significant.

In the warm period, the inverse association between the CA2 of temperature and AMI hospitalisations was slightly stronger than in the entire year (5% vs. 4%), without adjusting for any pollutant (Figure 1). Adjusting for O_3_ resulted in a weaker inverse association, and adjusting for PM_10_, NO_2_ and NO_x_ strengthened the inverse association somewhat to 5–7%. No association between the CA2 of temperature and AMI hospitalisations was noticeable in the cold period.

There was no association between the CA2 of temperature and IHD deaths in the entire year, warm or cold periods, with and without adjusting for any pollutant (Figure S6). Neither were there any significant associations between the pollutants and IHD deaths (Figure S7).

**Figure 2:**
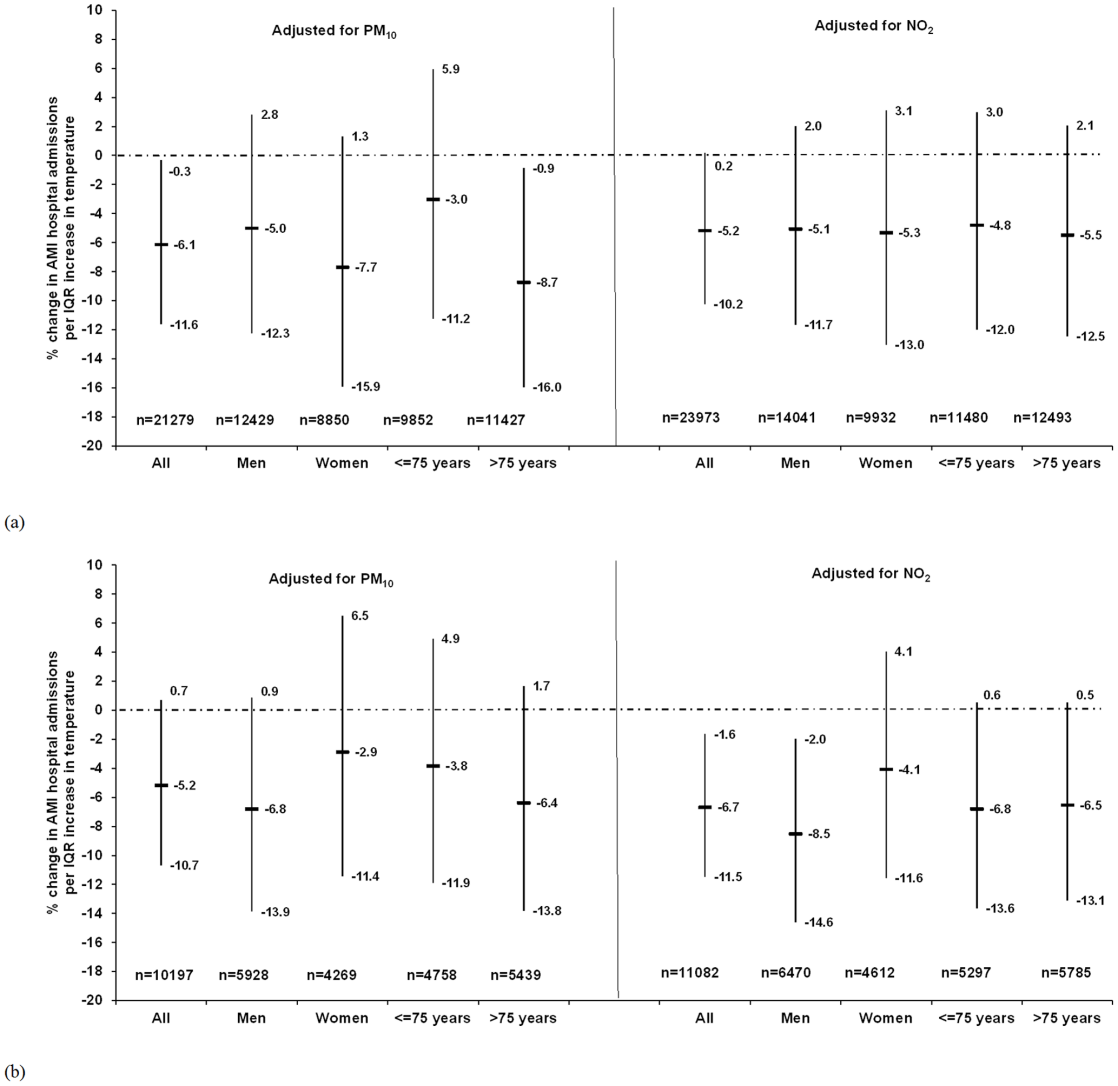
Association between temperature and acute myocardial infarction hospital admissions in Gothenburg by sex and age, expressed as percentage increase in risk (%) and 95% confidence intervals per inter-quartile increase in daily 2-day cumulative average during (a) the entire year and (b) warm period (April–September). Models adjusted for a single pollutant (same lag as temperature), relative humidity (same lag as temperature) and public holidays. Number of cases (n) used in the models is less than the original number due to missing exposure data p-values for the interaction term of CA2 of temperature and sex/age group were not significant (>0.05).

Figure 2 illustrates that stronger associations were observed between the CA2 of temperature and AMI hospitalisations for men and those older than 75 years (after adjustment for PM_10_), but the interaction terms were insignificant. No susceptible groups were identified in the warm period either, after adjusting for PM_10_ or NO_2_.

Inverse associations between temperature and AMI hospitalisations of similar effect size were in general observed in the case-crossover and GAM analyses (Table S2), with or without adjusting for PM_10_ or NO_2_.

## Discussion

In this case-crossover study from Gothenburg, Sweden, we observed a linear exposure-response relation corresponding to a 3% and 7% decrease in AMI hospitalisations for an IQR increase in the CA2 of temperature during the entire year (11°C) and the warm period (6°C), respectively, with and without adjustment for PM_10_, NO_2_, NO_x_ or O_3_. This translates into a 1–5% decrease in AMI hospitalisations per 1°C increase in the CA2 of temperature. We found no evidence of an association in the cold period or any association between temperature and IHD deaths in the entire year, warm or cold periods, with and without adjusting for any pollutant. No susceptible groups were identified either.

A review summarised the evidence of the association between temperature indices (e.g. daily mean, minimum and maximum temperature, diurnal temperature range, 3-hr maximum apparent temperature) and AMI hospitalisations [28]. Barnett et al concluded in a review that there is no single temperature index that is superior to others [29]. The observed effects in our study occurring within a day or two following exposure is compatible with other studies [6], [28]. The significant inverse association between daily mean temperature and AMI hospitalisations in Gothenburg (entire year and warm period) is in accordance with eight of 11 previous studies that reported significant effect estimates per unit change in a temperature index (i.e. not by categories of a temperature index) [13], [16], [17], [19]–[21], [23], [25]. These 11 studies were conducted in Europe, the USA and Asia. The strongest inverse association was observed for women in a time-series study from Trapani, Sicily (−9% per 1°C increase in minimum temperature) [19]. One study reported an insignificant inverse association between AMI hospitalisations in California and an increase in the daily apparent temperature (warm period) [22]. Two studies observed either an inverse or a positive association (not necessarily significant) between AMI hospitalisations with an increase in a temperature index, depending on whether the temperature index is minimum or maximum temperature and the location of the study (Los Angeles, San Francisco or Sacramento, USA) [15], [24]. The strongest positive association was observed for women (55–69 years) in Sacramento, California, USA (8% per 1°C increase in minimum temperature) [15]. Two of the 11 studies focused on the warm period (non-heat wave days) [22], [24] and one study investigated both warm and cold periods [25], whilst the rest investigated the entire year. The temperature ranged from −15°C in Lille, France to 38°C in California, USA. Although numerous ecological studies have shown a U-shape association between temperature and all types of CVD mortality or morbidity combined (i.e. all ICD10 codes I or all ICD9 codes 390–459) [30]–[34], most of the 11 studies on AMI confirmed a linear association. Only four of the studies investigated confounding by air pollution [20]–[22], [25].

Of the aforementioned studies, few investigated susceptible groups. Those that did, reported associations of the stratified analyses, but did not explicitly mention whether the interaction term between the susceptible variable and that of temperature was significant or not.

Apart from the general lack of investigating confounding by air pollution, other factors may explain the heterogeneity of risk estimates. These factors are related to the statistical methods used (different lags selected, lack of control for confounding by day of the week, public holidays and seasonality), the demographical profile of the study population (e.g. age, sex, socio-economic status), the efficiency and accessibility of the health system, diagnostic criteria of AMI, and type of hospitalisation (planned or emergency). Gothenburg had lower PM_10_ levels than the studies from Copenhagen, Denmark and Augsburg, Germany [20], [25]. Higher and lower O_3_ levels occurred in the city compared to those in Augsburg, Germany and California, USA, respectively [20], [22]. Slightly higher NO_2_ levels were observed in Gothenburg compared to those from Copenhagen, Denmark [25]. Other reasons for heterogeneity may be related to different PM composition, different indoor and outdoor air pollution sources, climate, behaviour and building traditions, which may influence indoor infiltration of outdoor air pollution and exposure to outdoor temperature [35].

Seasonal variation in CVD mortality and morbidity has been reported in the scientific literature since the early 1950s [36]. Some of this variation is dependent on temperature, as indicated in 11 other studies and ours, after controlling for seasons. Although we observed more AMI hospitalisations in the cold period, we did not observe any association between temperature and AMI hospitalisations in the cold period. A possible reason for the latter may be due to the widespread use of central heating in well insulated buildings in Sweden [35]. Most AMI hospitalisations also occurred among people older than 65 years and some of them may have restricted mobility and remain mostly indoors. Nevertheless, a study from Norway found that mean room temperature in the living room, kitchen and bathroom is about 20°C throughout the year in households of the elderly, but the bedroom temperature varies with the outdoor temperature as people sleep with a window slightly opened [37]. This behaviour is also common in Sweden. One likely mechanism behind the lower AMI morbidity and CVD mortality in warm seasons is vasodilation, resulting in more favourable cardiac hemodynamics [38]. Although the physiological mechanisms resulting from temperature changes to CVD outcomes that manifest at the clinical and public health scales (e.g. hospitalisations and deaths) are still not completely understood [28], vasoconstriction following a drop in ambient temperature is a likely mechanism. Other physiological markers along with blood pressure have a clear seasonal variation (increases in the colder months): red and white blood cell counts, blood viscosity, plasma cholesterol, coagulation factors (e.g. plasma fibrinogen, plasminogen activator inhibitor type 1) and inflammatory markers (e.g. IL-6, C-reactive protein) during winter [28].

The inverse association between outdoor temperature and AMI hospitalisations during the entire year in our study was driven by the inverse association in the warm period. This poses the question of the biological plausibility of such an association. Cold exposure studies (i.e. human volunteer studies) have also observed an increase in the afore-mentioned physiological factors with a decrease in temperature independent of season [39]. It is therefore possible that changes in these physiological markers for CVD may affect hospitalisations due to AMI, independent of season. Some of these physiological markers (e.g. blood pressure, heart rate, cardiac output and endothelial function) have a circadian rhythm [40]. It should be noted that the climate in Gothenburg in April to September is not hot; 25^th^ and 75^th^ percentiles 11 and 17°C with frequent rain and no heat waves. We therefore postulate that the physiological mechanisms behind the seasonality for AMI (higher risk during winter) may apply also in the “warm” season in countries with a mild climate in summer. In addition, people display different behaviour in diet, activity, housing (open windows), psychosocial factors and mood disorders on cold and warm days, and general well-being may be attenuated in cold and rainy days during summer [41], [42].

Our secondary aim was to examine associations between temperature and IHD deaths, but we did not find any such associations. The reason for this could be that IHD deaths that occurred out-of-hospital may have a lower diagnostic validity than the hospitalised AMI cases of the present study. Asymptomatic AMIs are not uncommon. Potentially, cold temperatures could precipitate chest pain symptoms, leading to hospitalisation and recognition of AMI, rather than influencing mechanisms, such as plaque rupture. However, in the absence of more detailed information on the episodes involved, this remains highly conjectural.

Although the focus of this study is on temperature, the lack of an association between AMI hospitalisations and IHD deaths with PM_10_, NO_2_ and O_3_ does warrant some discussion. Please see Text S1 for this discussion.

Advantages of our study include accurate meteorological, air pollution, AMI hospitalisation and IHD mortality data. Data from the Swedish Hospital Registry has been shown to have high validity for a diagnosis of MI [43]. Autopsy rates for persons dying outside hospital from IHD are high for younger but not older people [44]. Some disease misclassification is possible, but it is unlikely to be related to temperature. Our study period of 26 years is longer than the other studies that applied GAM analyses, which had study periods of 1–15 years [13], [15], [16], [18], [20], [21], [23], [25]. Unlike our study, most studies do not report the actual number of cases or days included in their case-crossover or GAM analyses, due to missing exposure data.

Another advantage of our study is that unlike most of the other studies, we investigated confounding by air pollution. Moreover, our results were robust regarding the association between temperature and AMI hospitalisations in the case-crossover and GAM analyses.

As with all ecological epidemiology study designs, our study has a disadvantage of exposure misclassification, i.e. the assumption that the ambient temperature, humidity and air pollution measured in the city are the same across Gothenburg. The exposure error resulting from using ambient temperature and air pollution as a surrogate for personal exposure can potentially lead to bias in the estimated association, and this can be more pronounced among the elderly and other frail groups who generally spend most of their time indoors.

Other limitations are the inability to adjust for PM_2.5_ (data available from 2006) and the lack of chemical composition of PM_10_ [45].

A third limitation is that information on effect modifiers, e.g. the use of medications, preexisting CVD or comorbidities [46]–[48], was not available in our study. Such effect modifiers may bias the association between the air pollutants and AMI hospital admissions in either direction.

In conclusion, our results support the notion that moderate increases in temperature are associated with a decrease in AMI hospitalisations in the entire year and warm period in a setting with no heat waves. This association (assumed to be causal) is complex and depends on the specific health outcome (death or hospitalisation), population characteristics (age, sex, socio-economic status), exposure conditions and the efficiency of the health care system, which all vary with time [49]. The International Panel on Climate Change stressed that many similar studies on temperature and health cannot be extrapolated infinitely into the future without considering major uncertainties regarding changes in populations, the rate and intensity of projected climate change and adaptation [50].

## Supporting Information

Figure S1 **Smoothed relationship (expressed as the model estimate) between acute myocardial infarction hospital admissions and a unit increase in daily 2-day cumulative average of (a) temperature and (b) relative humidity in Gothenburg, Sweden during the entire year (1985–2010).**
(DOCX)

Figure S2 **Time-series of acute myocardial infarction hospital admissions in Gothenburg, Sweden (1 January 1985–31 December 2010).**
(DOCX)

Figure S3 **Time-series of out-of-hospital ischemic heart disease deaths in Gothenburg, Sweden (1 January 1987–31 December 2010).**
(DOCX)

Figure S4 **Time-series of PM_10_ levels in Gothenburg, Sweden (1 January 1985–31 December 2010).**
(DOCX)

Figure S5 **Association between air pollutants and acute myocardial infarction hospital admissions in Gothenburg, expressed as percentage increase in risk (%) and 95% confidence intervals per inter-quartile increase in daily lag0, lag1 and 2-day cumulative average during (a) the entire year, (b) warm period (April−September) and (c) cold period (October−March).**
(DOCX)

Figure S6 **Association between temperature and out-of-hospital ischemic heart disease deaths in Gothenburg, expressed as percentage increase in risk (%) and 95% confidence intervals per inter-quartile increase in daily lag0, lag1 and 2-day cumulative average during (a) the entire year, (b) warm period (April−September) and (c) cold period (October−March).**
(DOCX)

Figure S7 **Association between air pollutants and out-of-hospital ischemic heart disease deaths in Gothenburg, expressed as percentage increase in risk (%) and 95% confidence intervals per inter-quartile increase in daily lag0, lag1 and 2-day cumulative average during (a) the entire year, (b) warm period (April−September) and (c) cold period (October−March).**
(DOCX)

Table S1 **Technical names of the various air pollution measurement instruments used in Gothenburg, Sweden during 1985–2010.**
(DOCX)

Table S2 **Association between temperature and acute myocardial infarction hospitalisations in Gothenburg, expressed as percentage increase in risk (%) and 95% confidence intervals per inter-quartile increase in the 2-day cumulative average (11°C).**
(DOCX)

Text S1 **Association between acute myocardial infarction hospitalisations and out-of-hospital ischemic heart disease deaths, and PM_10_, NO_2_ and O_3_ in Gothenburg, Sweden during 1985–2010.**
(DOCX)
