# Supplementary material for: Association between Ambient Temperature and Acute Myocardial Infarction Hospitalisations in Gothenburg, Sweden: 1985–2010
Source: PLoS One. 2013 Apr 30;8(4):e62059. doi: 10.1371/journal.pone.0062059 (PMC3639986; doi:10.1371/journal.pone.0062059)
Supplement: Figure S1 — Smoothed relationship (expressed as the model estimate) between acute myocardial infarction hospital admissions and a unit increase in daily 2-day cumulative average of (a) temperature and (b) relative humidity in Gothenburg, Sweden during the entire year (1985–2010). (DOCX) [file pone.0062059.s001.docx]

(a)

(b)

**Figure S1. Smoothed relationship (expressed as the model estimate) between acute myocardial infarction hospital admissions and a unit increase in daily 2-day cumulative average of (a) temperature and (b) relative humidity in Gothenburg, Sweden during the entire year (1985 − 2010).**

Models adjusted for daily 2-day cumulative average of relative humidity (or daily 2-day cumulative average of temperature), daily 2-day cumulative average of PM_10_, day of the week, public holidays and long-term trend (2.5 degrees of freedom/year).
