# Supplementary figures and images for: Association between Ambient Temperature and Acute Myocardial Infarction Hospitalisations in Gothenburg, Sweden: 1985–2010
Source: PLoS One. 2013 Apr 30;8(4):e62059. doi: 10.1371/journal.pone.0062059 (PMC3639986; doi:10.1371/journal.pone.0062059)

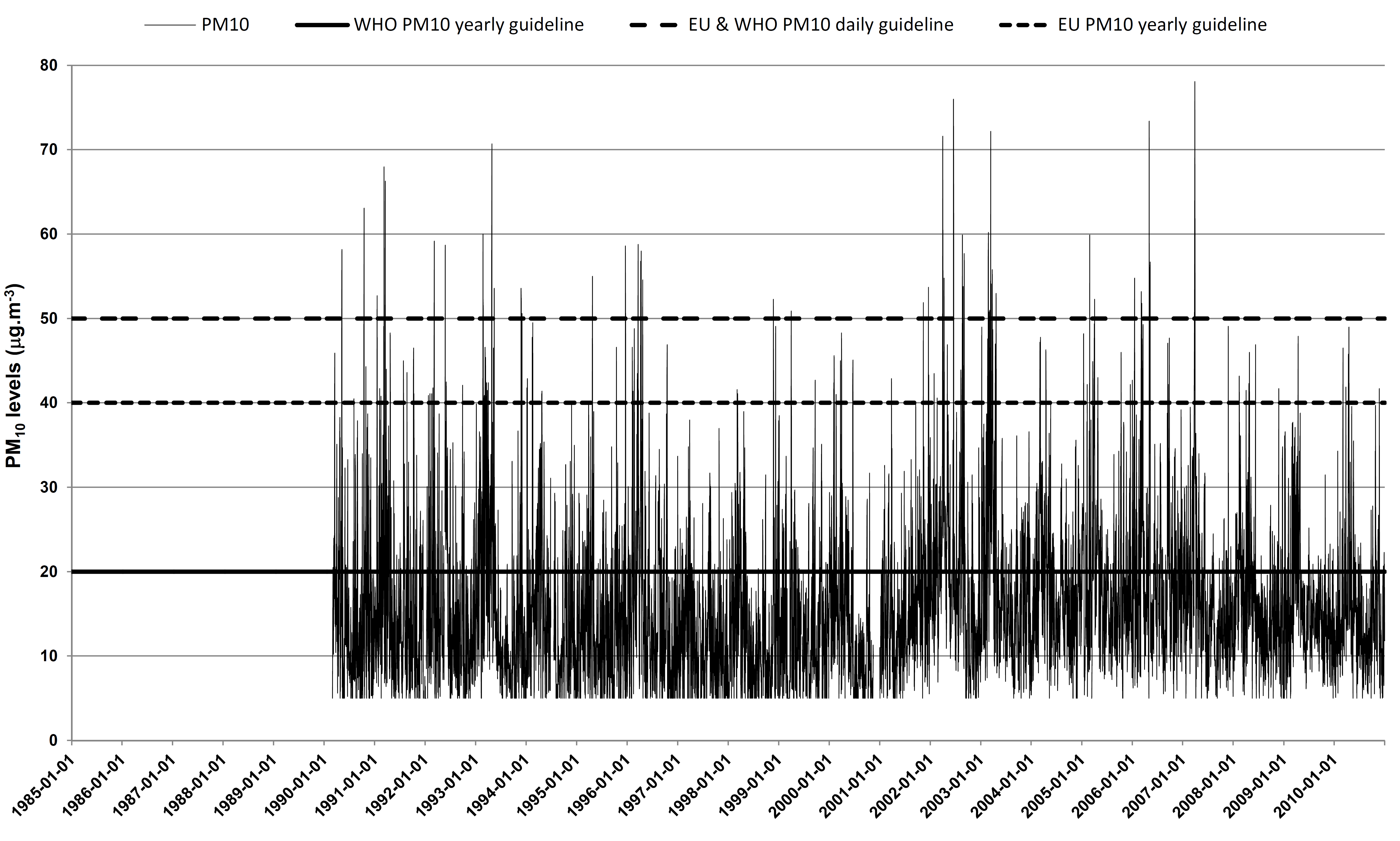


**Figure S4. Time-series of PM10 levels in Gothenburg, Sweden (1 January 1985 − 31 December 2010).**

Supplement: Figure S4 — Time-series of PM10 levels in Gothenburg, Sweden (1 January 1985–31 December 2010). (DOCX) [file pone.0062059.s004.docx]
