# Supplementary material for: Association between Ambient Temperature and Acute Myocardial Infarction Hospitalisations in Gothenburg, Sweden: 1985–2010
Source: PLoS One. 2013 Apr 30;8(4):e62059. doi: 10.1371/journal.pone.0062059 (PMC3639986; doi:10.1371/journal.pone.0062059)
Supplement: Figure S6 — Association between temperature and out-of-hospital ischemic heart disease deaths in Gothenburg, expressed as percentage increase in risk (%) and 95% confidence intervals per inter-quartile increase in daily lag0, lag1 and 2-day cumulative average during (a) the entire year, (b) warm period (April−September) and (c) cold period (October−March). (DOCX) [file pone.0062059.s006.docx]

(a)

(b)

(c)

**Figure S6. Association between temperature and out-of-hospital ischemic heart disease deaths in Gothenburg, expressed as percentage increase in risk (%) and 95% confidence intervals per inter-quartile increase in daily lag0, lag1 and 2-day cumulative average during (a) the entire year, (b) warm period (April-September) and (c) cold period (October-March).**

Models adjusted for a single pollutant (same lag as temperature), relative humidity (same lag as temperature) and public holidays

Number of cases (n) used in the models is less than the original number due to missing exposure data
