# Supplementary material for: Association between Ambient Temperature and Acute Myocardial Infarction Hospitalisations in Gothenburg, Sweden: 1985–2010
Source: PLoS One. 2013 Apr 30;8(4):e62059. doi: 10.1371/journal.pone.0062059 (PMC3639986; doi:10.1371/journal.pone.0062059)
Supplement: Table S1 — Technical names of the various air pollution measurement instruments used in Gothenburg, Sweden during 1985–2010. (DOCX) [file pone.0062059.s008.docx]

**Table S1. Technical names of the various air pollution measurement instruments used in Gothenburg, Sweden during 1985−2010**

| **Station name** | **Location** | **Air pollutant** | **Year** | **Instrument** | **Method** |
| --- | --- | --- | --- | --- | --- |
| Femman | Urban | PM_10_ | 1990−2010^a^ | TEOM 1400a | Tapered element oscillating microbalance |
| Femman | Urban | NO_X_/NO_2_ | 1985−1989 | Tecan CLD 502 | Chemiluminiscence |
| Femman | Urban | NO_X_/NO_2_ | 1989−2006 | Tecan CLD 700 AL | Chemiluminiscence |
| Femman | Urban | NO_X_/NO_2_ | 2006−2010 | Ecophysics CLD 700 AL | Chemiluminiscence |
| Råö | Rural | O_3_ | 1985−1995 | Monitor Labs 8810 | UV absorption |
| Råö | Rural | O_3_ | 1995−1997 | Monitor Labs 9810 | UV absorption |
| Råö | Rural | O_3_ | 1997−1998 | Dasibi 1008-AH/4655 | UV absorption |
| Råö | Rural | O_3_ | 1998−2001 | Monitor Labs 9810 | UV absorption |
| Råö | Rural | O_3_ | 2001−2002 | Thermo Electron 49C | UV absorption |
| Råö | Rural | O_3_ | 2002−2006 | Monitor Labs 9810 | UV absorption |
| Råö | Rural | O_3_ | 2006−2010 | Monitor Labs 8810 | UV absorption |

^a^No measurements during 1985−1989
