# Supplementary material for: Association between Ambient Temperature and Acute Myocardial Infarction Hospitalisations in Gothenburg, Sweden: 1985–2010
Source: PLoS One. 2013 Apr 30;8(4):e62059. doi: 10.1371/journal.pone.0062059 (PMC3639986; doi:10.1371/journal.pone.0062059)
Supplement: Table S2 — Association between temperature and acute myocardial infarction hospitalisations in Gothenburg, expressed as percentage increase in risk (%) and 95% confidence intervals per inter-quartile increase in the 2-day cumulative average (11°C). (DOCX) [file pone.0062059.s009.docx]

**Table S2. Association between temperature and acute myocardial infarction hospitalisations in Gothenburg, expressed as percentage increase in risk (%) and 95% confidence intervals per inter-quartile increase in the 2-day cumulative average (11 °C).**

| **Case-crossover analysis** | **n**^a^ | **%^b^** | **95% CI** | |
| --- | --- | --- | --- | --- |
| No pollutants | 25869 | -4.2 | -9.1 | 0.8 |
| Adjusted for PM_10_^c^ | 21279 | -6.1 | -11.6 | -0.3 |
| Adjusted for NO_2_^c^ | 23973 | -5.2 | -10.2 | 0.2 |
|  |  |  |  |  |
| **GAM analysis** | **n**^d^ | **%^e^** | **95% CI** | |
| No pollutants | 8828 | -6.4 | -10.7 | -1.8 |
| Adjusted for PM_10_^c^ | 7404 | -6.7 | -11.8 | -1.3 |
| Adjusted for NO_2_^c^ | 8216 | -6.8 | -11.4 | -2.0 |

^a^Number of cases used in the case-crossover analysis, which is less than 28 215 due to missing

exposure data

^b^Models adjusted for relative humidity (same lag as temperature) and public holidays

^c^Same lag as temperature

^d^Number of days used in the generalised additive Poisson time-series regression models,

which is less than 9496 days in the 26-year study period due to missing exposure data

^e^Models adjusted for relative humidity (same lag as temperature), public holidays, day

of the week and long-term trend (2.5 to 2.7 degrees of freedom/year)
