# Supplementary material for: Association between Ambient Temperature and Acute Myocardial Infarction Hospitalisations in Gothenburg, Sweden: 1985–2010
Source: PLoS One. 2013 Apr 30;8(4):e62059. doi: 10.1371/journal.pone.0062059 (PMC3639986; doi:10.1371/journal.pone.0062059)
Supplement: Text S1 — Association between acute myocardial infarction hospitalisations and out-of-hospital ischemic heart disease deaths, and PM10, NO2 and O3 in Gothenburg, Sweden during 1985–2010. (DOCX) [file pone.0062059.s010.docx]

**Text S1. Association between acute myocardial infarction hospitalisations and out-of-hospital ischemic heart disease deaths, and PM_10_, NO_2_ and O_3_ in Gothenburg, Sweden during 1985−2010.**

Bhaskaran et al and Mustafic et al concluded that the evidence suggests that ambient air pollution exposure is detrimental to AMI hospitalisation risk [1,2]. According to the meta-analysis by Mustafic et al, PM_2.5_ is the pollutant having the strongest association with AMI, with the risk increasing by 2.5% for each 10 μg.m^-3^ [1]. For PM_10_, NO_2_ and O_3_ the associations found are increases (per 10 μg.m^-3^) by 0.6%, 1.1% and 0.3%, respectively. Although NO_2_ levels were lower in the warm period in Gothenburg, we observed a tendency for AMI hospitalisations to increase by 2% per IQR (12 μg.m^-3^) increase in the CA2 of NO_2_, which translates into 1.4% per 10 μg.m^-3^ increase. Contrary to what is known about the toxicity of ground-level O_3_, AMI hospitalisations tended to decrease by 2% per IQR (19 μg.m^-3^) increase in the CA2 of O_3_, or -1.2% per 10 μg.m^-3^ increase. Twelve of the 19 studies included in the meta-analysis also reported inverse associations, but only 2 reported significant results. Studies use different exposure indices for O_3_, e.g. 8-hr mean (all year or warm months, but not clear on what is meant by 8-hr mean) or 24-hr mean (all year), and O_3_ levels recorded at urban and/or rural monitoring sites. Bhaskaran et al postulated reasons for the observed protective effects [2]. Higher O_3_ levels may be a proxy for reductions in other pollutant levels, especially if O_3_ is measured at an urban site. Mustafic et al also discussed possible reasons for the protective effects [1]. AMI is less prevalent in summer when O_3_ levels are higher. The mechanism of O_3_ formation is more closely dependent on solar radiation and brightness than temperature. In contrast to the warm period, where no obvious association was observed, an IQR (10 μg.m^-3^) increase in the CA2 of PM_10_ tended to increase AMI hospitalisations by 3% in the cold period, with borderline significance. No clear association was observed for the other pollutants in the cold period. PM_10_ levels were on average higher in the cold period and the chemical composition and toxicity may also be different in the two periods.

Although we found no significant association between any of the air pollutants and IHD deaths (i.e. severe cases), the effect estimate of 1.5% increase per 10 μg.m^-3^ increase in the CA2 of PM_10_ in the warm period is slightly higher than that of the latest meta-analyses (all types of CVD mortality, all year, 17 European studies): 0.9% [3,4]. Similarly, the effect estimate of 2% (4.6%) increase per 10 μg.m^-3^ (per IQR 23 μg.m^-3^) increase in the CA2 of O_3_ in the cold period is slightly higher than that of the latest meta-analysis (all types of CVD mortality, all year, 17 European studies, 8-hr mean of O_3_): 0.4% [3,4].

**References**

[1] Mustafic H, Jabre P, Caussin C, Murad MH, Escolano S, wt al (2012) Main air pollutants and myocardial infarction: a systematic review and meta-analysis. JAMA 307(7): 713−721.

[2] Bhaskaran K, Hajat S, Haines A, Herrett E, Wilkinson P, et al (2009) Effects of air pollution on the incidence of myocardial infarction. Heart 95(21): 1746−1759.

[3] Anderson HR, Atkinson RW, Peacock JL, Marston L, Konstantinou K (2004) Meta-Analysis of Time Series Studies and Panel Studies of Particulate Matter (PM) and Ozone (O_3_). World Health Organization: Copenhagen, Denmark. Available online: <http://www.euro.who.int/__data/assets/pdf_file/0004/74731/e82792.pdf> Accessed 14 November 2012.

[4] Anderson HR, Atkinson RW, Bremner SA, Carrington J, Peacock J (2007) Quantitative systematic review of short term associations between ambient air pollution (particulate matter, ozone, nitrogen dioxide, sulphur dioxide and carbon monoxide), and mortality and morbidity. Report to Department of Health revised following first review. Available online: <http://www.dh.gov.uk/en/Publicationsandstatistics/Publications/PublicationsPolicyAndGuidance/DH_121200>. Accessed 14 November 2012.
